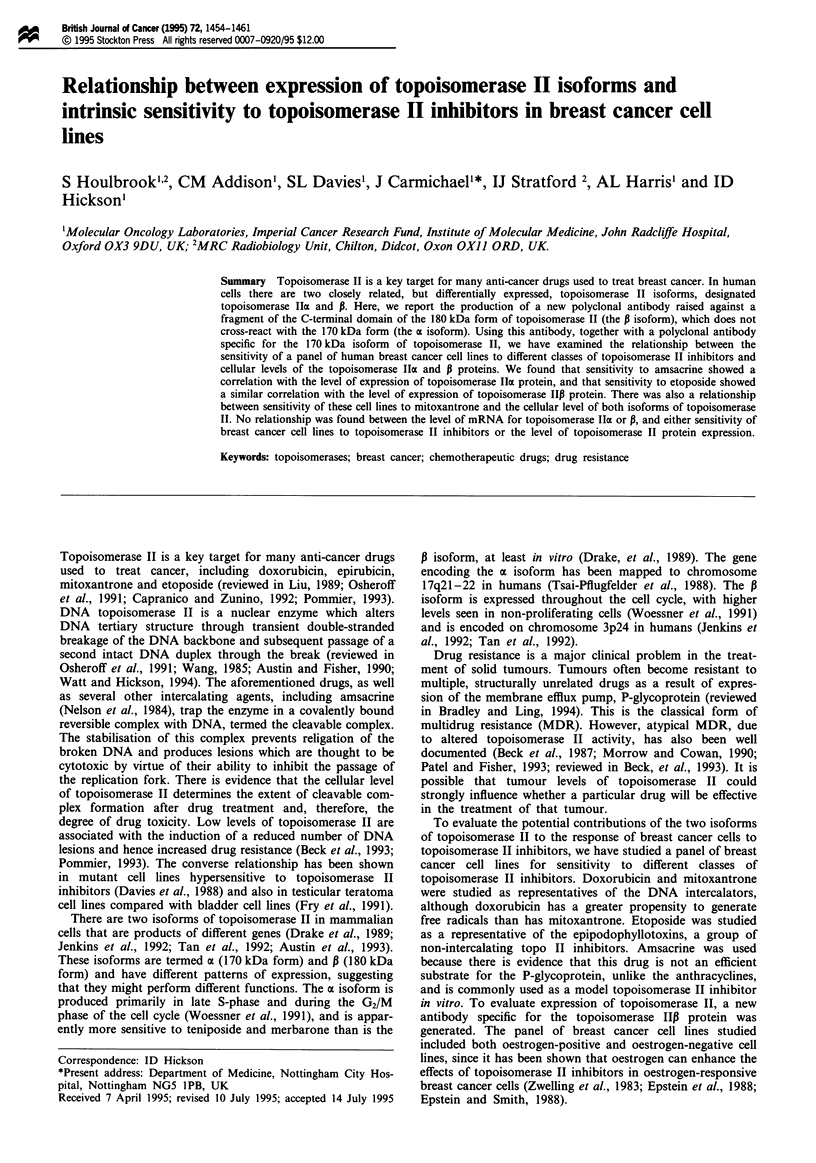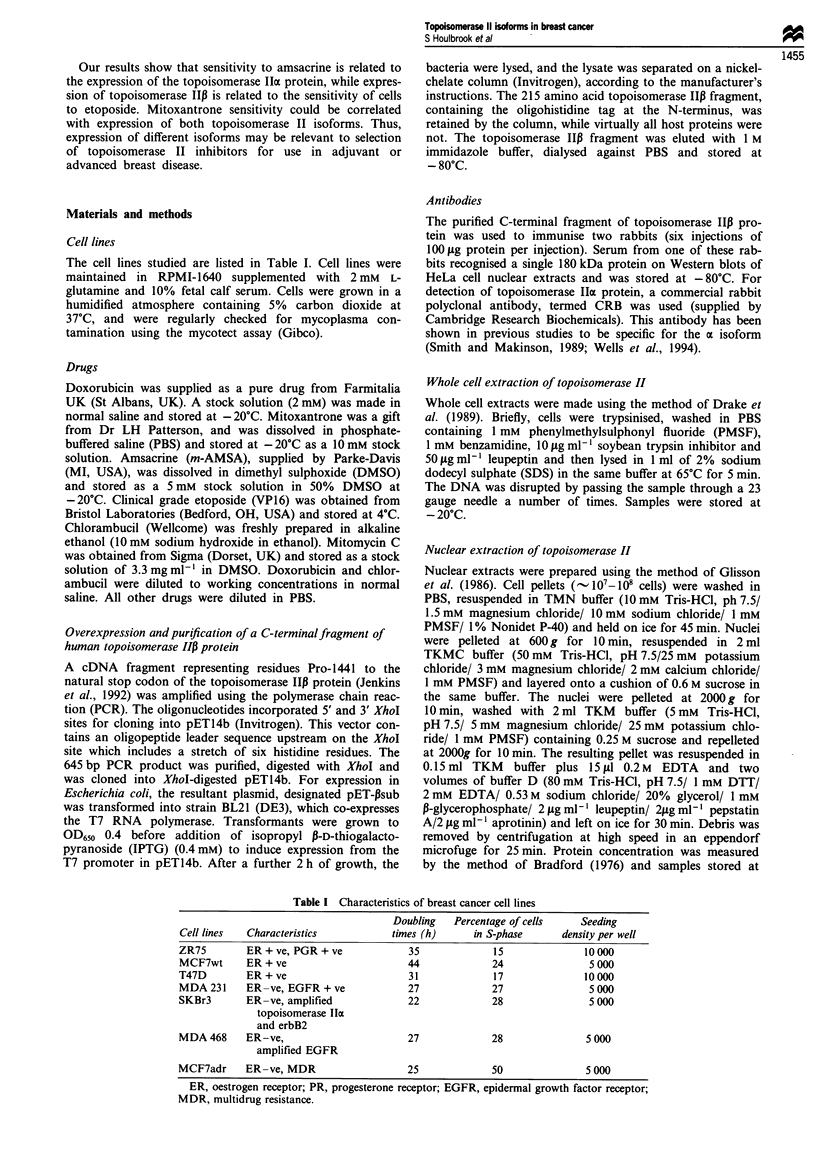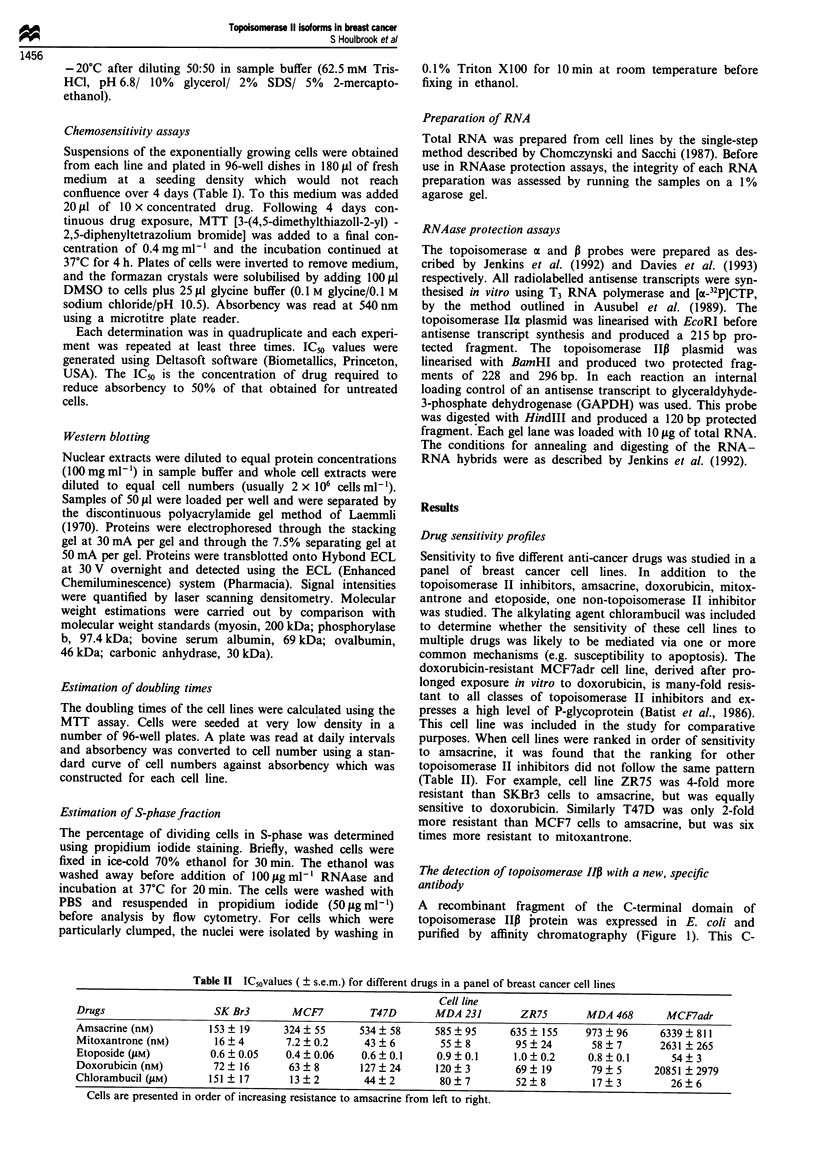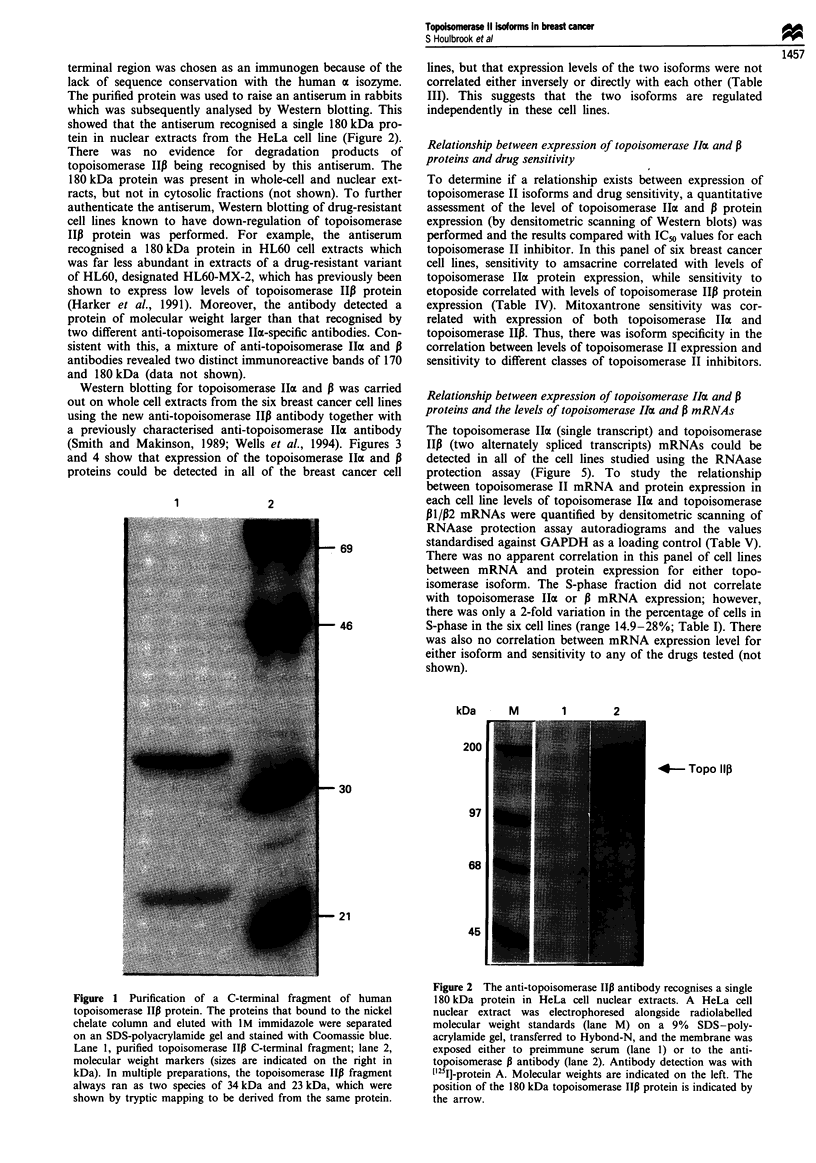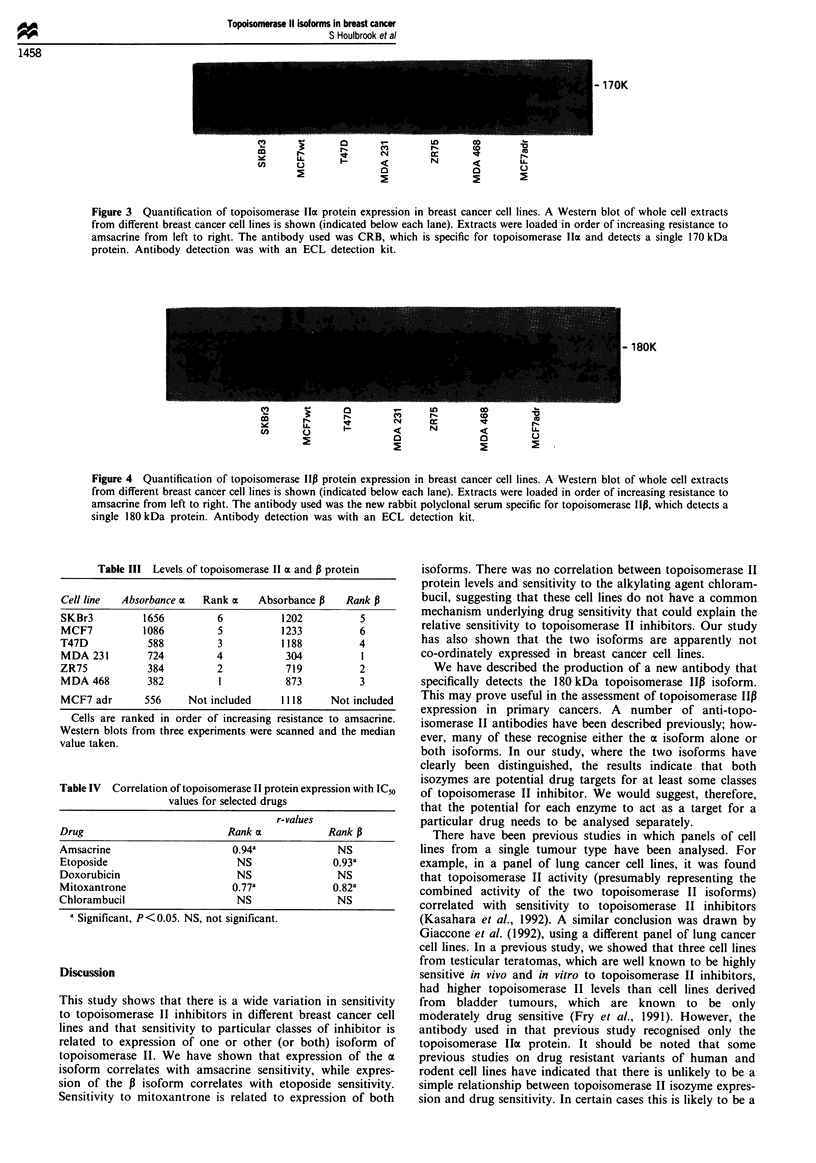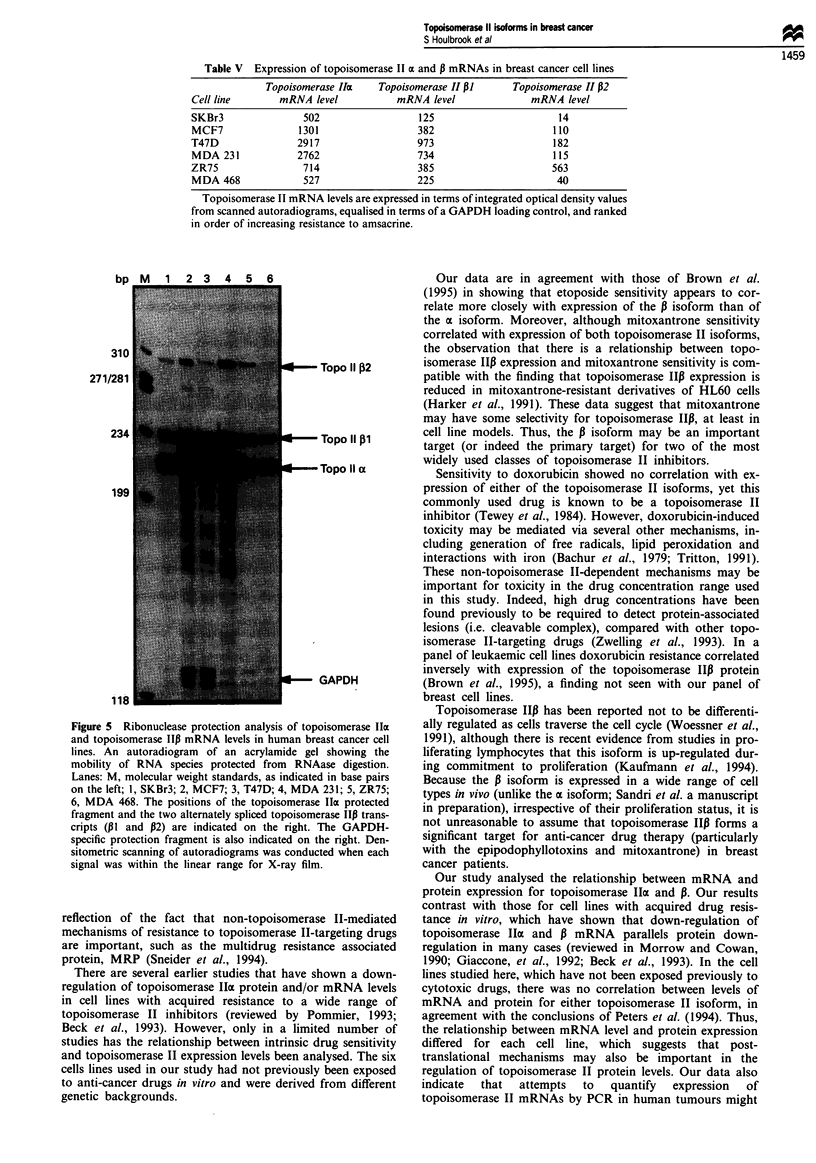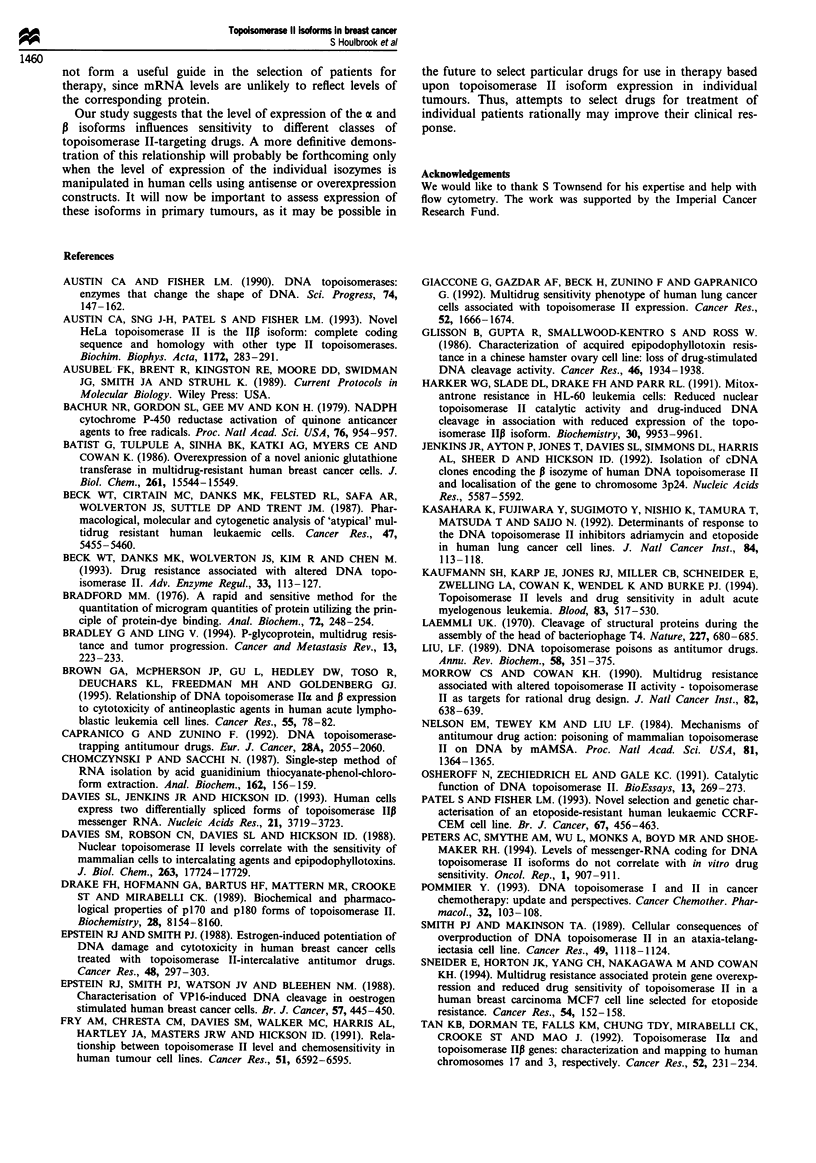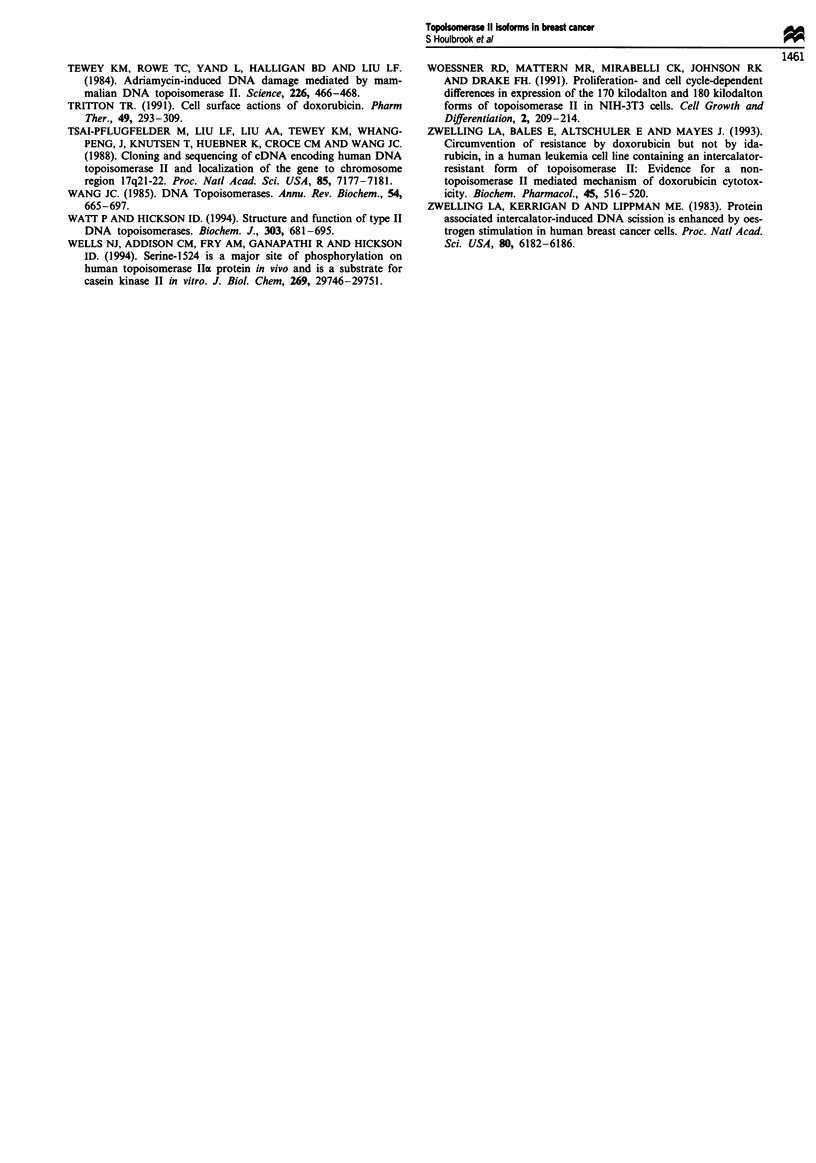# Relationship between expression of topoisomerase II isoforms and intrinsic sensitivity to topoisomerase II inhibitors in breast cancer cell lines.

**DOI:** 10.1038/bjc.1995.529

**Published:** 1995-12

**Authors:** S. Houlbrook, C. M. Addison, S. L. Davies, J. Carmichael, I. J. Stratford, A. L. Harris, I. D. Hickson

**Affiliations:** Molecular Oncology Laboratories, Imperial Cancer Research Fund, John Radcliffe Hospital, Oxford, UK.

## Abstract

**Images:**


British Journal of Cancer (1995) 72, 1454-1461
© 1995 Stockton Press All rights reserved 0007-0920/95 $12.00

### Relationship between expression of topoisomerase II isoforms and intrinsic sensitivity to topoisomerase II inhibitors in breast cancer cell lines

S Houlbrook[1,2], CM Addison[1], SL Davies[1], J Carmichael[1]*, IJ Stratford [2], AL Harris[1] and ID Hickson[1]

[1]*Molecular Oncology Laboratories, Imperial Cancer Research Fund, Institute of Molecular Medicine, John Radcliffe Hospital, Oxford OX3 9DU, UK;* [2]*MRC Radiobiology Unit, Chilton, Didcot, Oxon OX11 ORD, UK.*

**Summary** Topoisomerase II is a key target for many anti-cancer drugs used to treat breast cancer. In human cells there are two closely related, but differentially expressed, topoisomerase II isoforms, designated topoisomerase IIα and β. Here, we report the production of a new polyclonal antibody raised against a fragment of the C-terminal domain of the 180 kDa form of topoisomerase II (the β isoform), which does not cross-react with the 170 kDa form (the α isoform). Using this antibody, together with a polyclonal antibody specific for the 170 kDa isoform of topoisomerase II, we have examined the relationship between the sensitivity of a panel of human breast cancer cell lines to different classes of topoisomerase II inhibitors and cellular levels of the topoisomerase IIα and β proteins. We found that sensitivity to amsacrine showed a correlation with the level of expression of topoisomerase IIα protein, and that sensitivity to etoposide showed a similar correlation with the level of expression of topoisomerase IIβ protein. There was also a relationship between sensitivity of these cell lines to mitoxantrone and the cellular level of both isoforms of topoisomerase II. No relationship was found between the level of mRNA for topoisomerase IIα or β, and either sensitivity of breast cancer cell lines to topoisomerase II inhibitors or the level of topoisomerase II protein expression.

**Keywords:** topoisomerases; breast cancer; chemotherapeutic drugs; drug resistance

Topoisomerase II is a key target for many anti-cancer drugs used to treat cancer, including doxorubicin, epirubicin, mitoxantrone and etoposide (reviewed in Liu, 1989; Osheroff *et al.*, 1991; Capranico and Zunino, 1992; Pommier, 1993). DNA topoisomerase II is a nuclear enzyme which alters DNA tertiary structure through transient double-stranded breakage of the DNA backbone and subsequent passage of a second intact DNA duplex through the break (reviewed in Osheroff *et al.*, 1991; Wang, 1985; Austin and Fisher, 1990; Watt and Hickson, 1994). The aforementioned drugs, as well as several other intercalating agents, including amsacrine (Nelson *et al.*, 1984), trap the enzyme in a covalently bound reversible complex with DNA, termed the cleavable complex. The stabilisation of this complex prevents religation of the broken DNA and produces lesions which are thought to be cytotoxic by virtue of their ability to inhibit the passage of the replication fork. There is evidence that the cellular level of topoisomerase II determines the extent of cleavable complex formation after drug treatment and, therefore, the degree of drug toxicity. Low levels of topoisomerase II are associated with the induction of a reduced number of DNA lesions and hence increased drug resistance (Beck *et al.*, 1993; Pommier, 1993). The converse relationship has been shown in mutant cell lines hypersensitive to topoisomerase II inhibitors (Davies *et al.*, 1988) and also in testicular teratoma cell lines compared with bladder cell lines (Fry *et al.*, 1991).

There are two isoforms of topoisomerase II in mammalian cells that are products of different genes (Drake *et al.*, 1989; Jenkins *et al.*, 1992; Tan *et al.*, 1992; Austin *et al.*, 1993). These isoforms are termed α (170 kDa form) and β (180 kDa form) and have different patterns of expression, suggesting that they might perform different functions. The α isoform is produced primarily in late S-phase and during the $G_2/M$ phase of the cell cycle (Woessner *et al.*, 1991), and is apparently more sensitive to teniposide and merbarone than is the β isoform, at least *in vitro* (Drake, *et al.*, 1989). The gene encoding the α isoform has been mapped to chromosome 17q21-22 in humans (Tsai-Pflugfelder *et al.*, 1988). The β isoform is expressed throughout the cell cycle, with higher levels seen in non-proliferating cells (Woessner *et al.*, 1991) and is encoded on chromosome 3p24 in humans (Jenkins *et al.*, 1992; Tan *et al.*, 1992).

Drug resistance is a major clinical problem in the treatment of solid tumours. Tumours often become resistant to multiple, structurally unrelated drugs as a result of expression of the membrane efflux pump, P-glycoprotein (reviewed in Bradley and Ling, 1994). This is the classical form of multidrug resistance (MDR). However, atypical MDR, due to altered topoisomerase II activity, has also been well documented (Beck *et al.*, 1987; Morrow and Cowan, 1990; Patel and Fisher, 1993; reviewed in Beck, *et al.*, 1993). It is possible that tumour levels of topoisomerase II could strongly influence whether a particular drug will be effective in the treatment of that tumour.

To evaluate the potential contributions of the two isoforms of topoisomerase II to the response of breast cancer cells to topoisomerase II inhibitors, we have studied a panel of breast cancer cell lines for sensitivity to different classes of topoisomerase II inhibitors. Doxorubicin and mitoxantrone were studied as representatives of the DNA intercalators, although doxorubicin has a greater propensity to generate free radicals than has mitoxantrone. Etoposide was studied as a representative of the epipodophyllotoxins, a group of non-intercalating topo II inhibitors. Amsacrine was used because there is evidence that this drug is not an efficient substrate for the P-glycoprotein, unlike the anthracyclines, and is commonly used as a model topoisomerase II inhibitor *in vitro*. To evaluate expression of topoisomerase II, a new antibody specific for the topoisomerase IIβ protein was generated. The panel of breast cancer cell lines studied included both oestrogen-positive and oestrogen-negative cell lines, since it has been shown that oestrogen can enhance the effects of topoisomerase II inhibitors in oestrogen-responsive breast cancer cells (Zwelling *et al.*, 1983; Epstein *et al.*, 1988; Epstein and Smith, 1988).

Correspondence: ID Hickson
*Present address: Department of Medicine, Nottingham City Hospital, Nottingham NG5 1PB, UK
Received 7 April 1995; revised 10 July 1995; accepted 14 July 1995

Our results show that sensitivity to amsacrine is related to the expression of the topoisomerase IIα protein, while expression of topoisomerase IIβ is related to the sensitivity of cells to etoposide. Mitoxantrone sensitivity could be correlated with expression of both topoisomerase II isoforms. Thus, expression of different isoforms may be relevant to selection of topoisomerase II inhibitors for use in adjuvant or advanced breast disease.

### Materials and methods

### *Cell lines*

The cell lines studied are listed in Table I. Cell lines were maintained in RPMI-1640 supplemented with 2 mM L-glutamine and 10% fetal calf serum. Cells were grown in a humidified atmosphere containing 5% carbon dioxide at 37°C, and were regularly checked for mycoplasma contamination using the mycotect assay (Gibco).

### *Drugs*

Doxorubicin was supplied as a pure drug from Farmitalia UK (St Albans, UK). A stock solution (2 mM) was made in normal saline and stored at −20°C. Mitoxantrone was a gift from Dr LH Patterson, and was dissolved in phosphate-buffered saline (PBS) and stored at −20°C as a 10 mM stock solution. Amsacrine (*m*-AMSA), supplied by Parke-Davis (MI, USA), was dissolved in dimethyl sulphoxide (DMSO) and stored as a 5 mM stock solution in 50% DMSO at −20°C. Clinical grade etoposide (VP16) was obtained from Bristol Laboratories (Bedford, OH, USA) and stored at 4°C. Chlorambucil (Wellcome) was freshly prepared in alkaline ethanol (10 mM sodium hydroxide in ethanol). Mitomycin C was obtained from Sigma (Dorset, UK) and stored as a stock solution of 3.3 mg $ml^{-1}$ in DMSO. Doxorubicin and chlorambucil were diluted to working concentrations in normal saline. All other drugs were diluted in PBS.

### *Overexpression and purification of a C-terminal fragment of human topoisomerase IIβ protein*

A cDNA fragment representing residues Pro-1441 to the natural stop codon of the topoisomerase IIβ protein (Jenkins *et al.*, 1992) was amplified using the polymerase chain reaction (PCR). The oligonucleotides incorporated 5′ and 3′ *Xho*I sites for cloning into pET14b (Invitrogen). This vector contains an oligopeptide leader sequence upstream on the *Xho*I site which includes a stretch of six histidine residues. The 645 bp PCR product was purified, digested with *Xho*I and was cloned into *Xho*I-digested pET14b. For expression in *Escherichia coli*, the resultant plasmid, designated pET-βsub was transformed into strain BL21 (DE3), which co-expresses the T7 RNA polymerase. Transformants were grown to $OD_{650}$ 0.4 before addition of isopropyl β-D-thiogalactopyranoside (IPTG) (0.4 mM) to induce expression from the T7 promoter in pET14b. After a further 2 h of growth, the bacteria were lysed, and the lysate was separated on a nickel-chelate column (Invitrogen), according to the manufacturer's instructions. The 215 amino acid topoisomerase IIβ fragment, containing the oligohistidine tag at the N-terminus, was retained by the column, while virtually all host proteins were not. The topoisomerase IIβ fragment was eluted with 1 M immidazole buffer, dialysed against PBS and stored at −80°C.

### *Antibodies*

The purified C-terminal fragment of topoisomerase IIβ protein was used to immunise two rabbits (six injections of 100 μg protein per injection). Serum from one of these rabbits recognised a single 180 kDa protein on Western blots of HeLa cell nuclear extracts and was stored at −80°C. For detection of topoisomerase IIα protein, a commercial rabbit polyclonal antibody, termed CRB was used (supplied by Cambridge Research Biochemicals). This antibody has been shown in previous studies to be specific for the α isoform (Smith and Makinson, 1989; Wells *et al.*, 1994).

### *Whole cell extraction of topoisomerase II*

Whole cell extracts were made using the method of Drake *et al.* (1989). Briefly, cells were trypsinised, washed in PBS containing 1 mM phenylmethylsulphonyl fluoride (PMSF), 1 mM benzamidine, 10 μg $ml^{-1}$ soybean trypsin inhibitor and 50 μg $ml^{-1}$ leupeptin and then lysed in 1 ml of 2% sodium dodecyl sulphate (SDS) in the same buffer at 65°C for 5 min. The DNA was disrupted by passing the sample through a 23 gauge needle a number of times. Samples were stored at −20°C.

### *Nuclear extraction of topoisomerase II*

Nuclear extracts were prepared using the method of Glisson *et al.* (1986). Cell pellets ($\sim 10^7 - 10^8$ cells) were washed in PBS, resuspended in TMN buffer (10 mM Tris-HCl, ph 7.5/ 1.5 mM magnesium chloride/ 10 mM sodium chloride/ 1 mM PMSF/ 1% Nonidet P-40) and held on ice for 45 min. Nuclei were pelleted at 600 *g* for 10 min, resuspended in 2 ml TKMC buffer (50 mM Tris-HCl, pH 7.5/25 mM potassium chloride/ 3 mM magnesium chloride/ 2 mM calcium chloride/ 1 mM PMSF) and layered onto a cushion of 0.6 M sucrose in the same buffer. The nuclei were pelleted at 2000 *g* for 10 min, washed with 2 ml TKM buffer (5 mM Tris-HCl, pH 7.5/ 5 mM magnesium chloride/ 25 mM potassium chloride/ 1 mM PMSF) containing 0.25 M sucrose and repelleted at 2000*g* for 10 min. The resulting pellet was resuspended in 0.15 ml TKM buffer plus 15 μl 0.2 M EDTA and two volumes of buffer D (80 mM Tris-HCl, pH 7.5/ 1 mM DTT/ 2 mM EDTA/ 0.53 M sodium chloride/ 20% glycerol/ 1 mM β-glycerophosphate/ 2 μg $ml^{-1}$ leupeptin/ 2μg $ml^{-1}$ pepstatin A/2 μg $ml^{-1}$ aprotinin) and left on ice for 30 min. Debris was removed by centrifugation at high speed in an eppendorf microfuge for 25 min. Protein concentration was measured by the method of Bradford (1976) and samples stored at

**Table I** Characteristics of breast cancer cell lines

| *Cell lines* | *Characteristics* | *Doubling times (h)* | *Percentage of cells in S-phase* | *Seeding density per well* |
|---|---|---|---|---|
| ZR75 | ER + ve, PGR + ve | 35 | 15 | 10 000 |
| MCF7wt | ER + ve | 44 | 24 | 5 000 |
| T47D | ER + ve | 31 | 17 | 10 000 |
| MDA 231 | ER − ve, EGFR + ve | 27 | 27 | 5 000 |
| SKBr3 | ER − ve, amplified topoisomerase IIα and erbB2 | 22 | 28 | 5 000 |
| MDA 468 | ER − ve, amplified EGFR | 27 | 28 | 5 000 |
| MCF7adr | ER − ve, MDR | 25 | 50 | 5 000 |

ER, oestrogen receptor; PR, progesterone receptor; EGFR, epidermal growth factor receptor; MDR, multidrug resistance.

−20°C after diluting 50:50 in sample buffer (62.5 mM Tris-HCl, pH 6.8/ 10% glycerol/ 2% SDS/ 5% 2-mercapto-ethanol).

*Chemosensitivity assays*

Suspensions of the exponentially growing cells were obtained from each line and plated in 96-well dishes in 180 μl of fresh medium at a seeding density which would not reach confluence over 4 days (Table I). To this medium was added 20 μl of 10 × concentrated drug. Following 4 days continuous drug exposure, MTT [3-(4,5-dimethylthiazoll-2-yl) - 2,5-diphenyltetrazolium bromide] was added to a final concentration of 0.4 mg $ml^{-1}$ and the incubation continued at 37°C for 4 h. Plates of cells were inverted to remove medium, and the formazan crystals were solubilised by adding 100 μl DMSO to cells plus 25 μl glycine buffer (0.1 M glycine/0.1 M sodium chloride/pH 10.5). Absorbency was read at 540 nm using a microtitre plate reader.

Each determination was in quadruplicate and each experiment was repeated at least three times. $IC_{50}$ values were generated using Deltasoft software (Biometallics, Princeton, USA). The $IC_{50}$ is the concentration of drug required to reduce absorbency to 50% of that obtained for untreated cells.

*Western blotting*

Nuclear extracts were diluted to equal protein concentrations (100 mg $ml^{-1}$) in sample buffer and whole cell extracts were diluted to equal cell numbers (usually $2 \times 10^6$ cells $ml^{-1}$). Samples of 50 μl were loaded per well and were separated by the discontinuous polyacrylamide gel method of Laemmli (1970). Proteins were electrophoresed through the stacking gel at 30 mA per gel and through the 7.5% separating gel at 50 mA per gel. Proteins were transblotted onto Hybond ECL at 30 V overnight and detected using the ECL (Enhanced Chemiluminescence) system (Pharmacia). Signal intensities were quantified by laser scanning densitometry. Molecular weight estimations were carried out by comparison with molecular weight standards (myosin, 200 kDa; phosphorylase b, 97.4 kDa; bovine serum albumin, 69 kDa; ovalbumin, 46 kDa; carbonic anhydrase, 30 kDa).

*Estimation of doubling times*

The doubling times of the cell lines were calculated using the MTT assay. Cells were seeded at very low density in a number of 96-well plates. A plate was read at daily intervals and absorbency was converted to cell number using a standard curve of cell numbers against absorbency which was constructed for each cell line.

*Estimation of S-phase fraction*

The percentage of dividing cells in S-phase was determined using propidium iodide staining. Briefly, washed cells were fixed in ice-cold 70% ethanol for 30 min. The ethanol was washed away before addition of 100 μg $ml^{-1}$ RNAase and incubation at 37°C for 20 min. The cells were washed with PBS and resuspended in propidium iodide (50 μg $ml^{-1}$) before analysis by flow cytometry. For cells which were particularly clumped, the nuclei were isolated by washing in 0.1% Triton X100 for 10 min at room temperature before fixing in ethanol.

*Preparation of RNA*

Total RNA was prepared from cell lines by the single-step method described by Chomczynski and Sacchi (1987). Before use in RNAase protection assays, the integrity of each RNA preparation was assessed by running the samples on a 1% agarose gel.

*RNAase protection assays*

The topoisomerase α and β probes were prepared as described by Jenkins *et al.* (1992) and Davies *et al.* (1993) respectively. All radiolabelled antisense transcripts were synthesised *in vitro* using $T_3$ RNA polymerase and [α-$^{32}$P]CTP, by the method outlined in Ausubel *et al.* (1989). The topoisomerase IIα plasmid was linearised with *Eco*RI before antisense transcript synthesis and produced a 215 bp protected fragment. The topoisomerase IIβ plasmid was linearised with *Bam*HI and produced two protected fragments of 228 and 296 bp. In each reaction an internal loading control of an antisense transcript to glyceraldyhyde-3-phosphate dehydrogenase (GAPDH) was used. This probe was digested with *Hin*dIII and produced a 120 bp protected fragment. Each gel lane was loaded with 10 μg of total RNA. The conditions for annealing and digesting of the RNA–RNA hybrids were as described by Jenkins *et al.* (1992).

### Results

*Drug sensitivity profiles*

Sensitivity to five different anti-cancer drugs was studied in a panel of breast cancer cell lines. In addition to the topoisomerase II inhibitors, amsacrine, doxorubicin, mitoxantrone and etoposide, one non-topoisomerase II inhibitor was studied. The alkylating agent chlorambucil was included to determine whether the sensitivity of these cell lines to multiple drugs was likely to be mediated via one or more common mechanisms (e.g. susceptibility to apoptosis). The doxorubicin-resistant MCF7adr cell line, derived after prolonged exposure *in vitro* to doxorubicin, is many-fold resistant to all classes of topoisomerase II inhibitors and expresses a high level of P-glycoprotein (Batist *et al.*, 1986). This cell line was included in the study for comparative purposes. When cell lines were ranked in order of sensitivity to amsacrine, it was found that the ranking for other topoisomerase II inhibitors did not follow the same pattern (Table II). For example, cell line ZR75 was 4-fold more resistant than SKBr3 cells to amsacrine, but was equally sensitive to doxorubicin. Similarly T47D was only 2-fold more resistant than MCF7 cells to amsacrine, but was six times more resistant to mitoxantrone.

*The detection of topoisomerase IIβ with a new, specific antibody*

A recombinant fragment of the C-terminal domain of topoisomerase IIβ protein was expressed in *E. coli* and purified by affinity chromatography (Figure 1). This C-

**Table II** $IC_{50}$values ( ± s.e.m.) for different drugs in a panel of breast cancer cell lines

| | Cell line | | | | | | |
|---|---|---|---|---|---|---|---|
| *Drugs* | *SK Br3* | *MCF7* | *T47D* | *MDA 231* | *ZR75* | *MDA 468* | *MCF7adr* |
| Amsacrine (nM) | 153 ± 19 | 324 ± 55 | 534 ± 58 | 585 ± 95 | 635 ± 155 | 973 ± 96 | 6339 ± 811 |
| Mitoxantrone (nM) | 16 ± 4 | 7.2 ± 0.2 | 43 ± 6 | 55 ± 8 | 95 ± 24 | 58 ± 7 | 2631 ± 265 |
| Etoposide (μM) | 0.6 ± 0.05 | 0.4 ± 0.06 | 0.6 ± 0.1 | 0.9 ± 0.1 | 1.0 ± 0.2 | 0.8 ± 0.1 | 54 ± 3 |
| Doxorubicin (nM) | 72 ± 16 | 63 ± 8 | 127 ± 24 | 120 ± 3 | 69 ± 19 | 79 ± 5 | 20851 ± 2979 |
| Chlorambucil (μM) | 151 ± 17 | 13 ± 2 | 44 ± 2 | 80 ± 7 | 52 ± 8 | 17 ± 3 | 26 ± 6 |

Cells are presented in order of increasing resistance to amsacrine from left to right.

terminal region was chosen as an immunogen because of the lack of sequence conservation with the human α isozyme. The purified protein was used to raise an antiserum in rabbits which was subsequently analysed by Western blotting. This showed that the antiserum recognised a single 180 kDa protein in nuclear extracts from the HeLa cell line (Figure 2). There was no evidence for degradation products of topoisomerase IIβ being recognised by this antiserum. The 180 kDa protein was present in whole-cell and nuclear extracts, but not in cytosolic fractions (not shown). To further authenticate the antiserum, Western blotting of drug-resistant cell lines known to have down-regulation of topoisomerase IIβ protein was performed. For example, the antiserum recognised a 180 kDa protein in HL60 cell extracts which was far less abundant in extracts of a drug-resistant variant of HL60, designated HL60-MX-2, which has previously been shown to express low levels of topoisomerase IIβ protein (Harker *et al.*, 1991). Moreover, the antibody detected a protein of molecular weight larger than that recognised by two different anti-topoisomerase IIα-specific antibodies. Consistent with this, a mixture of anti-topoisomerase IIα and β antibodies revealed two distinct immunoreactive bands of 170 and 180 kDa (data not shown).

Western blotting for topoisomerase IIα and β was carried out on whole cell extracts from the six breast cancer cell lines using the new anti-topoisomerase IIβ antibody together with a previously characterised anti-topoisomerase IIα antibody (Smith and Makinson, 1989; Wells *et al.*, 1994). Figures 3 and 4 show that expression of the topoisomerase IIα and β proteins could be detected in all of the breast cancer cell

**Figure 1** Purification of a C-terminal fragment of human topoisomerase IIβ protein. The proteins that bound to the nickel chelate column and eluted with 1M immidazole were separated on an SDS-polyacrylamide gel and stained with Coomassie blue. Lane 1, purified topoisomerase IIβ C-terminal fragment; lane 2, molecular weight markers (sizes are indicated on the right in kDa). In multiple preparations, the topoisomerase IIβ fragment always ran as two species of 34 kDa and 23 kDa, which were shown by tryptic mapping to be derived from the same protein.

lines, but that expression levels of the two isoforms were not correlated either inversely or directly with each other (Table III). This suggests that the two isoforms are regulated independently in these cell lines.

*Relationship between expression of topoisomerase IIα and β proteins and drug sensitivity*

To determine if a relationship exists between expression of topoisomerase II isoforms and drug sensitivity, a quantitative assessment of the level of topoisomerase IIα and β protein expression (by densitometric scanning of Western blots) was performed and the results compared with $IC_{50}$ values for each topoisomerase II inhibitor. In this panel of six breast cancer cell lines, sensitivity to amsacrine correlated with levels of topoisomerase IIα protein expression, while sensitivity to etoposide correlated with levels of topoisomerase IIβ protein expression (Table IV). Mitoxantrone sensitivity was correlated with expression of both topoisomerase IIα and topoisomerase IIβ. Thus, there was isoform specificity in the correlation between levels of topoisomerase II expression and sensitivity to different classes of topoisomerase II inhibitors.

*Relationship between expression of topoisomerase IIα and β proteins and the levels of topoisomerase IIα and β mRNAs*

The topoisomerase IIα (single transcript) and topoisomerase IIβ (two alternately spliced transcripts) mRNAs could be detected in all of the cell lines studied using the RNAase protection assay (Figure 5). To study the relationship between topoisomerase II mRNA and protein expression in each cell line levels of topoisomerase IIα and topoisomerase β1/β2 mRNAs were quantified by densitometric scanning of RNAase protection assay autoradiograms and the values standardised against GAPDH as a loading control (Table V). There was no apparent correlation in this panel of cell lines between mRNA and protein expression for either topoisomerase isoform. The S-phase fraction did not correlate with topoisomerase IIα or β mRNA expression; however, there was only a 2-fold variation in the percentage of cells in S-phase in the six cell lines (range 14.9–28%; Table I). There was also no correlation between mRNA expression level for either isoform and sensitivity to any of the drugs tested (not shown).

**Figure 2** The anti-topoisomerase IIβ antibody recognises a single 180 kDa protein in HeLa cell nuclear extracts. A HeLa cell nuclear extract was electrophoresed alongside radiolabelled molecular weight standards (lane M) on a 9% SDS–polyacrylamide gel, transferred to Hybond-N, and the membrane was exposed either to preimmune serum (lane 1) or to the anti-topoisomerase β antibody (lane 2). Antibody detection was with $[^{125}I]$-protein A. Molecular weights are indicated on the left. The position of the 180 kDa topoisomerase IIβ protein is indicated by the arrow.

**Figure 3** Quantification of topoisomerase IIα protein expression in breast cancer cell lines. A Western blot of whole cell extracts from different breast cancer cell lines is shown (indicated below each lane). Extracts were loaded in order of increasing resistance to amsacrine from left to right. The antibody used was CRB, which is specific for topoisomerase IIα and detects a single 170 kDa protein. Antibody detection was with an ECL detection kit.

**Figure 4** Quantification of topoisomerase IIβ protein expression in breast cancer cell lines. A Western blot of whole cell extracts from different breast cancer cell lines is shown (indicated below each lane). Extracts were loaded in order of increasing resistance to amsacrine from left to right. The antibody used was the new rabbit polyclonal serum specific for topoisomerase IIβ, which detects a single 180 kDa protein. Antibody detection was with an ECL detection kit.

**Table III** Levels of topoisomerase II α and β protein

| *Cell line* | *Absorbance* α | Rank α | Absorbance β | *Rank* β |
|---|---|---|---|---|
| SKBr3 | 1656 | 6 | 1202 | 5 |
| MCF7 | 1086 | 5 | 1233 | 6 |
| T47D | 588 | 3 | 1188 | 4 |
| MDA 231 | 724 | 4 | 304 | 1 |
| ZR75 | 384 | 2 | 719 | 2 |
| MDA 468 | 382 | 1 | 873 | 3 |
| MCF7 adr | 556 | Not included | 1118 | Not included |

Cells are ranked in order of increasing resistance to amsacrine. Western blots from three experiments were scanned and the median value taken.

**Table IV** Correlation of topoisomerase II protein expression with $IC_{50}$ values for selected drugs

| | *r-values* | |
|---|---|---|
| *Drug* | *Rank* α | *Rank* β |
| Amsacrine | 0.94[a] | NS |
| Etoposide | NS | 0.93[a] |
| Doxorubicin | NS | NS |
| Mitoxantrone | 0.77[a] | 0.82[a] |
| Chlorambucil | NS | NS |

[a] Significant, $P<0.05$. NS, not significant.

### Discussion

This study shows that there is a wide variation in sensitivity to topoisomerase II inhibitors in different breast cancer cell lines and that sensitivity to particular classes of inhibitor is related to expression of one or other (or both) isoform of topoisomerase II. We have shown that expression of the α isoform correlates with amsacrine sensitivity, while expression of the β isoform correlates with etoposide sensitivity. Sensitivity to mitoxantrone is related to expression of both isoforms. There was no correlation between topoisomerase II protein levels and sensitivity to the alkylating agent chlorambucil, suggesting that these cell lines do not have a common mechanism underlying drug sensitivity that could explain the relative sensitivity to topoisomerase II inhibitors. Our study has also shown that the two isoforms are apparently not co-ordinately expressed in breast cancer cell lines.

We have described the production of a new antibody that specifically detects the 180 kDa topoisomerase IIβ isoform. This may prove useful in the assessment of topoisomerase IIβ expression in primary cancers. A number of anti-topoisomerase II antibodies have been described previously; however, many of these recognise either the α isoform alone or both isoforms. In our study, where the two isoforms have clearly been distinguished, the results indicate that both isozymes are potential drug targets for at least some classes of topoisomerase II inhibitor. We would suggest, therefore, that the potential for each enzyme to act as a target for a particular drug needs to be analysed separately.

There have been previous studies in which panels of cell lines from a single tumour type have been analysed. For example, in a panel of lung cancer cell lines, it was found that topoisomerase II activity (presumably representing the combined activity of the two topoisomerase II isoforms) correlated with sensitivity to topoisomerase II inhibitors (Kasahara *et al.*, 1992). A similar conclusion was drawn by Giaccone *et al.* (1992), using a different panel of lung cancer cell lines. In a previous study, we showed that three cell lines from testicular teratomas, which are well known to be highly sensitive *in vivo* and *in vitro* to topoisomerase II inhibitors, had higher topoisomerase II levels than cell lines derived from bladder tumours, which are known to be only moderately drug sensitive (Fry *et al.*, 1991). However, the antibody used in that previous study recognised only the topoisomerase IIα protein. It should be noted that some previous studies on drug resistant variants of human and rodent cell lines have indicated that there is unlikely to be a simple relationship between topoisomerase II isozyme expression and drug sensitivity. In certain cases this is likely to be a

**Table V** Expression of topoisomerase II α and β mRNAs in breast cancer cell lines

| Cell line | Topoisomerase IIα mRNA level | Topoisomerase II β1 mRNA level | Topoisomerase II β2 mRNA level |
|---|---|---|---|
| SKBr3 | 502 | 125 | 14 |
| MCF7 | 1301 | 382 | 110 |
| T47D | 2917 | 973 | 182 |
| MDA 231 | 2762 | 734 | 115 |
| ZR75 | 714 | 385 | 563 |
| MDA 468 | 527 | 225 | 40 |

Topoisomerase II mRNA levels are expressed in terms of integrated optical density values from scanned autoradiograms, equalised in terms of a GAPDH loading control, and ranked in order of increasing resistance to amsacrine.

**Figure 5** Ribonuclease protection analysis of topoisomerase IIα and topoisomerase IIβ mRNA levels in human breast cancer cell lines. An autoradiogram of an acrylamide gel showing the mobility of RNA species protected from RNAase digestion. Lanes: M, molecular weight standards, as indicated in base pairs on the left; 1, SKBr3; 2, MCF7; 3, T47D; 4, MDA 231; 5, ZR75; 6, MDA 468. The positions of the topoisomerase IIα protected fragment and the two alternately spliced topoisomerase IIβ transcripts (β1 and β2) are indicated on the right. The GAPDH-specific protection fragment is also indicated on the right. Densitometric scanning of autoradiograms was conducted when each signal was within the linear range for X-ray film.

reflection of the fact that non-topoisomerase II-mediated mechanisms of resistance to topoisomerase II-targeting drugs are important, such as the multidrug resistance associated protein, MRP (Sneider *et al.*, 1994).

There are several earlier studies that have shown a down-regulation of topoisomerase IIα protein and/or mRNA levels in cell lines with acquired resistance to a wide range of topoisomerase II inhibitors (reviewed by Pommier, 1993; Beck *et al.*, 1993). However, only in a limited number of studies has the relationship between intrinsic drug sensitivity and topoisomerase II expression levels been analysed. The six cells lines used in our study had not previously been exposed to anti-cancer drugs *in vitro* and were derived from different genetic backgrounds.

Our data are in agreement with those of Brown *et al.* (1995) in showing that etoposide sensitivity appears to correlate more closely with expression of the β isoform than of the α isoform. Moreover, although mitoxantrone sensitivity correlated with expression of both topoisomerase II isoforms, the observation that there is a relationship between topoisomerase IIβ expression and mitoxantrone sensitivity is compatible with the finding that topoisomerase IIβ expression is reduced in mitoxantrone-resistant derivatives of HL60 cells (Harker *et al.*, 1991). These data suggest that mitoxantrone may have some selectivity for topoisomerase IIβ, at least in cell line models. Thus, the β isoform may be an important target (or indeed the primary target) for two of the most widely used classes of topoisomerase II inhibitors.

Sensitivity to doxorubicin showed no correlation with expression of either of the topoisomerase II isoforms, yet this commonly used drug is known to be a topoisomerase II inhibitor (Tewey *et al.*, 1984). However, doxorubicin-induced toxicity may be mediated via several other mechanisms, including generation of free radicals, lipid peroxidation and interactions with iron (Bachur *et al.*, 1979; Tritton, 1991). These non-topoisomerase II-dependent mechanisms may be important for toxicity in the drug concentration range used in this study. Indeed, high drug concentrations have been found previously to be required to detect protein-associated lesions (i.e. cleavable complex), compared with other topoisomerase II-targeting drugs (Zwelling *et al.*, 1993). In a panel of leukaemic cell lines doxorubicin resistance correlated inversely with expression of the topoisomerase IIβ protein (Brown *et al.*, 1995), a finding not seen with our panel of breast cell lines.

Topoisomerase IIβ has been reported not to be differentially regulated as cells traverse the cell cycle (Woessner *et al.*, 1991), although there is recent evidence from studies in proliferating lymphocytes that this isoform is up-regulated during commitment to proliferation (Kaufmann *et al.*, 1994). Because the β isoform is expressed in a wide range of cell types *in vivo* (unlike the α isoform; Sandri *et al.* a manuscript in preparation), irrespective of their proliferation status, it is not unreasonable to assume that topoisomerase IIβ forms a significant target for anti-cancer drug therapy (particularly with the epipodophyllotoxins and mitoxantrone) in breast cancer patients.

Our study analysed the relationship between mRNA and protein expression for topoisomerase IIα and β. Our results contrast with those for cell lines with acquired drug resistance *in vitro*, which have shown that down-regulation of topoisomerase IIα and β mRNA parallels protein down-regulation in many cases (reviewed in Morrow and Cowan, 1990; Giaccone, *et al.*, 1992; Beck *et al.*, 1993). In the cell lines studied here, which have not been exposed previously to cytotoxic drugs, there was no correlation between levels of mRNA and protein for either topoisomerase II isoform, in agreement with the conclusions of Peters *et al.* (1994). Thus, the relationship between mRNA level and protein expression differed for each cell line, which suggests that post-translational mechanisms may also be important in the regulation of topoisomerase II protein levels. Our data also indicate that attempts to quantify expression of topoisomerase II mRNAs by PCR in human tumours might not form a useful guide in the selection of patients for therapy, since mRNA levels are unlikely to reflect levels of the corresponding protein.

Our study suggests that the level of expression of the α and β isoforms influences sensitivity to different classes of topoisomerase II-targeting drugs. A more definitive demonstration of this relationship will probably be forthcoming only when the level of expression of the individual isozymes is manipulated in human cells using antisense or overexpression constructs. It will now be important to assess expression of these isoforms in primary tumours, as it may be possible in the future to select particular drugs for use in therapy based upon topoisomerase II isoform expression in individual tumours. Thus, attempts to select drugs for treatment of individual patients rationally may improve their clinical response.

**Acknowledgements**
We would like to thank S Townsend for his expertise and help with flow cytometry. The work was supported by the Imperial Cancer Research Fund.

**References**

AUSTIN CA AND FISHER LM. (1990). DNA topoisomerases: enzymes that change the shape of DNA. *Sci. Progress*, **74**, 147-162.

AUSTIN CA, SNG J-H, PATEL S AND FISHER LM. (1993). Novel HeLa topoisomerase II is the IIβ isoform: complete coding sequence and homology with other type II topoisomerases. *Biochim. Biophys. Acta*, **1172**, 283-291.

AUSUBEL FK, BRENT R, KINGSTON RE, MOORE DD, SWIDMAN JG, SMITH JA AND STRUHL K. (1989). *Current Protocols in Molecular Biology*. Wiley Press: USA.

BACHUR NR, GORDON SL, GEE MV AND KON H. (1979). NADPH cytochrome P-450 reductase activation of quinone anticancer agents to free radicals. *Proc. Natl Acad. Sci. USA*, **76**, 954-957.

BATIST G, TULPULE A, SINHA BK, KATKI AG, MYERS CE AND COWAN K. (1986). Overexpression of a novel anionic glutathione transferase in multidrug-resistant human breast cancer cells. *J. Biol. Chem.*, **261**, 15544-15549.

BECK WT, CIRTAIN MC, DANKS MK, FELSTED RL, SAFA AR, WOLVERTON JS, SUTTLE DP AND TRENT JM. (1987). Pharmacological, molecular and cytogenetic analysis of 'atypical' multidrug resistant human leukaemic cells. *Cancer Res.*, **47**, 5455-5460.

BECK WT, DANKS MK, WOLVERTON JS, KIM R AND CHEN M. (1993). Drug resistance associated with altered DNA topoisomerase II. *Adv. Enzyme Regul.*, **33**, 113-127.

BRADFORD MM. (1976). A rapid and sensitive method for the quantitation of microgram quantities of protein utilizing the principle of protein-dye binding. *Anal. Biochem.*, **72**, 248-254.

BRADLEY G AND LING V. (1994). P-glycoprotein, multidrug resistance and tumor progression. *Cancer and Metastasis Rev.*, **13**, 223-233.

BROWN GA, McPHERSON JP, GU L, HEDLEY DW, TOSO R, DEUCHARS KL, FREEDMAN MH AND GOLDENBERG GJ. (1995). Relationship of DNA topoisomerase IIα and β expression to cytotoxicity of antineoplastic agents in human acute lymphoblastic leukemia cell lines. *Cancer Res.*, **55**, 78-82.

CAPRANICO G AND ZUNINO F. (1992). DNA topoisomerase-trapping antitumour drugs. *Eur. J. Cancer*, **28A**, 2055-2060.

CHOMCZYNSKI P AND SACCHI N. (1987). Single-step method of RNA isolation by acid guanidinium thiocyanate-phenol-chloroform extraction. *Anal. Biochem.*, **162**, 156-159.

DAVIES SL, JENKINS JR AND HICKSON ID. (1993). Human cells express two differentially spliced forms of topoisomerase IIβ messenger RNA. *Nucleic Acids Res.*, **21**, 3719-3723.

DAVIES SM, ROBSON CN, DAVIES SL AND HICKSON ID. (1988). Nuclear topoisomerase II levels correlate with the sensitivity of mammalian cells to intercalating agents and epipodophyllotoxins. *J. Biol. Chem.*, **263**, 17724-17729.

DRAKE FH, HOFMANN GA, BARTUS HF, MATTERN MR, CROOKE ST AND MIRABELLI CK. (1989). Biochemical and pharmacological properties of p170 and p180 forms of topoisomerase II. *Biochemistry*, **28**, 8154-8160.

EPSTEIN RJ AND SMITH PJ. (1988). Estrogen-induced potentiation of DNA damage and cytotoxicity in human breast cancer cells treated with topoisomerase II-intercalative antitumor drugs. *Cancer Res.*, **48**, 297-303.

EPSTEIN RJ, SMITH PJ, WATSON JV AND BLEEHEN NM. (1988). Characterisation of VP16-induced DNA cleavage in oestrogen stimulated human breast cancer cells. *Br. J. Cancer*, **57**, 445-450.

FRY AM, CHRESTA CM, DAVIES SM, WALKER MC, HARRIS AL, HARTLEY JA, MASTERS JRW AND HICKSON ID. (1991). Relationship between topoisomerase II level and chemosensitivity in human tumour cell lines. *Cancer Res.*, **51**, 6592-6595.

GIACCONE G, GAZDAR AF, BECK H, ZUNINO F AND GAPRANICO G. (1992). Multidrug sensitivity phenotype of human lung cancer cells associated with topoisomerase II expression. *Cancer Res.*, **52**, 1666-1674.

GLISSON B, GUPTA R, SMALLWOOD-KENTRO S AND ROSS W. (1986). Characterization of acquired epipodophyllotoxin resistance in a chinese hamster ovary cell line: loss of drug-stimulated DNA cleavage activity. *Cancer Res.*, **46**, 1934-1938.

HARKER WG, SLADE DL, DRAKE FH AND PARR RL. (1991). Mitoxantrone resistance in HL-60 leukemia cells: Reduced nuclear topoisomerase II catalytic activity and drug-induced DNA cleavage in association with reduced expression of the topoisomerase IIβ isoform. *Biochemistry*, **30**, 9953-9961.

JENKINS JR, AYTON P, JONES T, DAVIES SL, SIMMONS DL, HARRIS AL, SHEER D AND HICKSON ID. (1992). Isolation of cDNA clones encoding the β isozyme of human DNA topoisomerase II and localisation of the gene to chromosome 3p24. *Nucleic Acids Res.*, 5587-5592.

KASAHARA K, FUJIWARA Y, SUGIMOTO Y, NISHIO K, TAMURA T, MATSUDA T AND SAIJO N. (1992). Determinants of response to the DNA topoisomerase II inhibitors adriamycin and etoposide in human lung cancer cell lines. *J. Natl Cancer Inst.*, **84**, 113-118.

KAUFMANN SH, KARP JE, JONES RJ, MILLER CB, SCHNEIDER E, ZWELLING LA, COWAN K, WENDEL K AND BURKE PJ. (1994). Topoisomerase II levels and drug sensitivity in adult acute myelogenous leukemia. *Blood*, **83**, 517-530.

LAEMMLI UK. (1970). Cleavage of structural proteins during the assembly of the head of bacteriophage T4. *Nature*, **227**, 680-685.

LIU, LF. (1989). DNA topoisomerase poisons as antitumor drugs. *Annu. Rev. Biochem.*, **58**, 351-375.

MORROW CS AND COWAN KH. (1990). Multidrug resistance associated with altered topoisomerase II activity - topoisomerase II as targets for rational drug design. *J. Natl Cancer Inst.*, **82**, 638-639.

NELSON EM, TEWEY KM AND LIU LF. (1984). Mechanisms of antitumour drug action: poisoning of mammalian topoisomerase II on DNA by mAMSA. *Proc. Natl Acad. Sci. USA*, **81**, 1364-1365.

OSHEROFF N, ZECHIEDRICH EL AND GALE KC. (1991). Catalytic function of DNA topoisomerase II. *BioEssays*, **13**, 269-273.

PATEL S AND FISHER LM. (1993). Novel selection and genetic characterisation of an etoposide-resistant human leukaemic CCRF-CEM cell line. *Br. J. Cancer*, **67**, 456-463.

PETERS AC, SMYTHE AM, WU L, MONKS A, BOYD MR AND SHOEMAKER RH. (1994). Levels of messenger-RNA coding for DNA topoisomerase II isoforms do not correlate with *in vitro* drug sensitivity. *Oncol. Rep.*, **1**, 907-911.

POMMIER Y. (1993). DNA topoisomerase I and II in cancer chemotherapy: update and perspectives. *Cancer Chemother. Pharmacol.*, **32**, 103-108.

SMITH PJ AND MAKINSON TA. (1989). Cellular consequences of overproduction of DNA topoisomerase II in an ataxia-telangiectasia cell line. *Cancer Res.*, **49**, 1118-1124.

SNEIDER E, HORTON JK, YANG CH, NAKAGAWA M AND COWAN KH. (1994). Multidrug resistance associated protein gene overexpression and reduced drug sensitivity of topoisomerase II in a human breast carcinoma MCF7 cell line selected for etoposide resistance. *Cancer Res.*, **54**, 152-158.

TAN KB, DORMAN TE, FALLS KM, CHUNG TDY, MIRABELLI CK, CROOKE ST AND MAO J. (1992). Topoisomerase IIα and topoisomerase IIβ genes: characterization and mapping to human chromosomes 17 and 3, respectively. *Cancer Res.*, **52**, 231-234.

TEWEY KM, ROWE TC, YAND L, HALLIGAN BD AND LIU LF. (1984). Adriamycin-induced DNA damage mediated by mammalian DNA topoisomerase II. *Science*, **226**, 466–468.

TRITTON TR. (1991). Cell surface actions of doxorubicin. *Pharm Ther.*, **49**, 293–309.

TSAI-PFLUGFELDER M, LIU LF, LIU AA, TEWEY KM, WHANG-PENG, J, KNUTSEN T, HUEBNER K, CROCE CM AND WANG JC. (1988). Cloning and sequencing of cDNA encoding human DNA topoisomerase II and localization of the gene to chromosome region 17q21-22. *Proc. Natl Acad. Sci. USA*, **85**, 7177–7181.

WANG JC. (1985). DNA Topoisomerases. *Annu. Rev. Biochem.*, **54**, 665–697.

WATT P AND HICKSON ID. (1994). Structure and function of type II DNA topoisomerases. *Biochem. J.*, **303**, 681–695.

WELLS NJ, ADDISON CM, FRY AM, GANAPATHI R AND HICKSON ID. (1994). Serine-1524 is a major site of phosphorylation on human topoisomerase IIα protein *in vivo* and is a substrate for casein kinase II *in vitro*. *J. Biol. Chem*, **269**, 29746–29751.

WOESSNER RD, MATTERN MR, MIRABELLI CK, JOHNSON RK AND DRAKE FH. (1991). Proliferation- and cell cycle-dependent differences in expression of the 170 kilodalton and 180 kilodalton forms of topoisomerase II in NIH-3T3 cells. *Cell Growth and Differentiation*, **2**, 209–214.

ZWELLING LA, BALES E, ALTSCHULER E AND MAYES J. (1993). Circumvention of resistance by doxorubicin but not by idarubicin, in a human leukemia cell line containing an intercalator-resistant form of topoisomerase II: Evidence for a non-topoisomerase II mediated mechanism of doxorubicin cytotoxicity. *Biochem. Pharmacol.*, **45**, 516–520.

ZWELLING LA, KERRIGAN D AND LIPPMAN ME. (1983). Protein associated intercalator-induced DNA scission is enhanced by oestrogen stimulation in human breast cancer cells. *Proc. Natl Acad. Sci. USA*, **80**, 6182–6186.