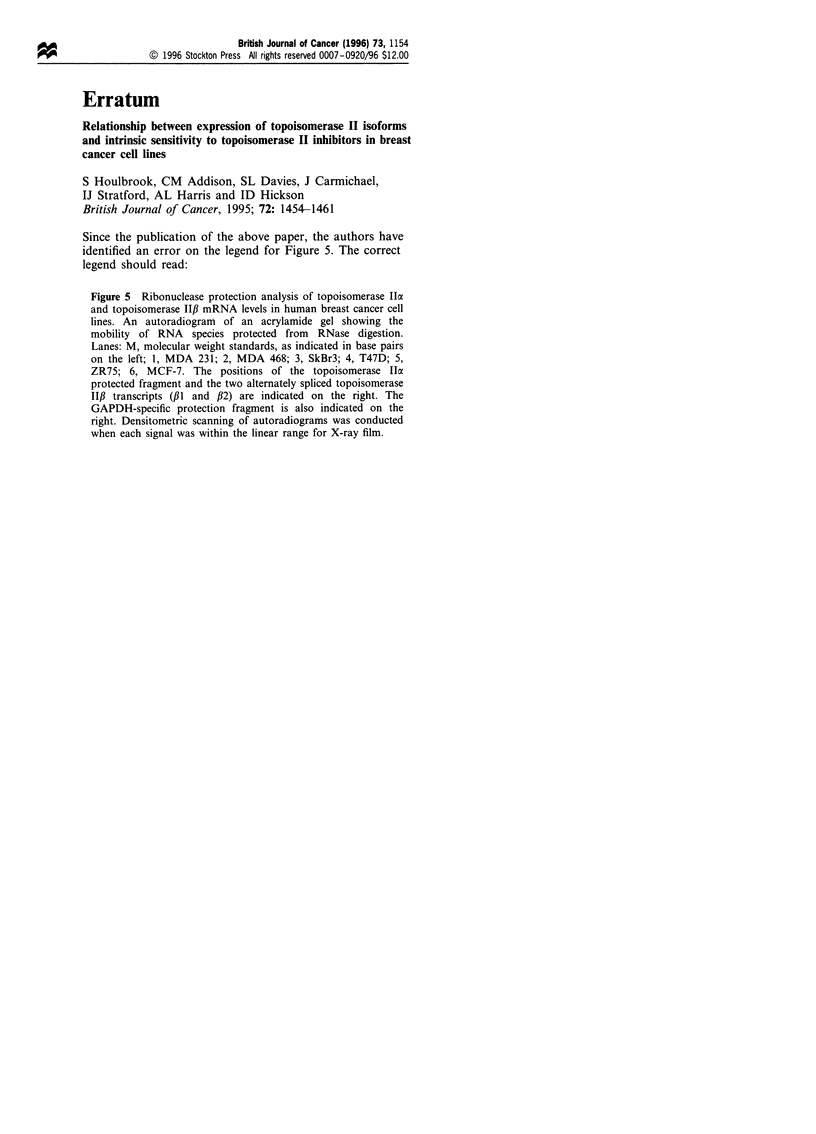# Relationship between expression of topoisomerase II isoforms and intrinsic sensitivity to topoisomerase II inhibitors in breast cancer cell lines

**Published:** 1996-10

**Authors:** 


British Journal of Cancer (1996) 73, 1154
© 1996 Stockton Press All rights reserved 0007-0920/96 $12.00

### Erratum

**Relationship between expression of topoisomerase II isoforms and intrinsic sensitivity to topoisomerase II inhibitors in breast cancer cell lines**

S Houlbrook, CM Addison, SL Davies, J Carmichael, IJ Stratford, AL Harris and ID Hickson
*British Journal of Cancer*, 1995; **72:** 1454–1461

Since the publication of the above paper, the authors have identified an error on the legend for Figure 5. The correct legend should read:

**Figure 5** Ribonuclease protection analysis of topoisomerase II$\alpha$ and topoisomerase II$\beta$ mRNA levels in human breast cancer cell lines. An autoradiogram of an acrylamide gel showing the mobility of RNA species protected from RNase digestion. Lanes: M, molecular weight standards, as indicated in base pairs on the left; 1, MDA 231; 2, MDA 468; 3, SkBr3; 4, T47D; 5, ZR75; 6, MCF-7. The positions of the topoisomerase II$\alpha$ protected fragment and the two alternately spliced topoisomerase II$\beta$ transcripts ($\beta$1 and $\beta$2) are indicated on the right. The GAPDH-specific protection fragment is also indicated on the right. Densitometric scanning of autoradiograms was conducted when each signal was within the linear range for X-ray film.